# A Review of the Theoretical Basis, Effects, and Cost Effectiveness of Online Smoking Cessation Interventions in the Netherlands: A Mixed-Methods Approach

**DOI:** 10.2196/jmir.7209

**Published:** 2017-06-23

**Authors:** Kei Long Cheung, Ben Wijnen, Hein de Vries

**Affiliations:** ^1^ Department of Health Services Research Maastricht University Maastricht Netherlands; ^2^ Department of Health Promotion Maastricht University Maastricht Netherlands

**Keywords:** Smoking cessation, telemedicine, review, online intervention, Internet-based intervention, behavioral change techniques, Netherlands

## Abstract

**Background:**

Tobacco smoking is a worldwide public health problem. In 2015, 26.3% of the Dutch population aged 18 years and older smoked, 74.4% of them daily. More and more people have access to the Internet worldwide; approximately 94% of the Dutch population have online access. Internet-based smoking cessation interventions (online cessation interventions) provide an opportunity to tackle the scourge of tobacco.

**Objective:**

The goal of this paper was to provide an overview of online cessation interventions in the Netherlands, while exploring their effectivity, cost effectiveness, and theoretical basis.

**Methods:**

A mixed-methods approach was used to identify Dutch online cessation interventions, using (1) a scientific literature search, (2) a grey literature search, and (3) expert input. For the scientific literature, the Cochrane review was used and updated by two independent researchers (n=651 identified studies), screening titles, abstracts, and then full-text studies between 2013 and 2016 (CENTRAL, MEDLINE, and EMBASE). For the grey literature, the researchers conducted a Google search (n=100 websites), screening for titles and first pages. Including expert input, this resulted in six interventions identified in the scientific literature and 39 interventions via the grey literature. Extracted data included effectiveness, cost effectiveness, theoretical factors, and behavior change techniques used.

**Results:**

Overall, many interventions (45 identified) were offered. Of the 45 that we identified, only six that were included in trials provided data on effectiveness. Four of these were shown to be effective and cost effective. In the scientific literature, 83% (5/6) of these interventions included changing attitudes, providing social support, increasing self-efficacy, motivating smokers to make concrete action plans to prepare their attempts to quit and to cope with challenges, supporting identity change and advising on changing routines, coping, and medication use. In all, 50% (3/6) of the interventions included a reward for abstinence. Interventions identified in the grey literature were less consistent, with inclusion of each theoretical factor ranging from 31% to 67% and of each behavior change technique ranging from 28% to 54%.

**Conclusions:**

Although the Internet may provide the opportunity to offer various smoking cessation programs, the user is left bewildered as far as efficacy is concerned, as most of these data are not available nor offered to the smokers. Clear regulations about the effectiveness of these interventions need to be devised to avoid disappointment and failed quitting attempts. Thus, there is a need for policy regulations to regulate the proliferation of these interventions and to foster their quality in the Netherlands.

## Introduction

### Tobacco Smoking

Tobacco smoking is a worldwide public health problem, with more than 5 million deaths being attributable to direct smoking [1]. Nonsmokers are also impacted by tobacco smoke as a result of second-hand smoke (passive smoking); an estimated 600,000 deaths are caused by smoking behavior, which affects various related diseases such as lung cancer, heart diseases, and chronic obstructive pulmonary disease. In the Netherlands, smoking is a public health problem. In 2015, 26.3% of the Dutch population aged 18 years and older smoked, 74.4% of them daily [2]. Of all smokers, 15.6% were considered heavy smokers, meaning that these individuals smoke an average of 20 or more cigarettes per day [2]. Estimates showed that in 2013, 19,000 deaths were attributable to smoking-related diseases in the Netherlands [3].

### Online Smoking Cessation Interventions

In an attempt to tackle the scourge of tobacco smoking, many smoking cessation interventions have been developed and proven effective, especially the more intensive interventions such as one-to-one behavioral therapy [4]. Each intervention has strengths and limitations. The more intensive interventions are often expensive, inconvenient to the recipient (eg, waiting list and the need to take time off work), and reach only a small proportion of smokers [5]. The Internet provides opportunities to address the smoking problem, as the Internet has grown to be an extremely important medium and is embedded in daily life in the Dutch population (and many other parts of the world). The opportunities provided by the Internet to enhance smoking cessation have led to the development of Internet-based interventions. Internet-based smoking cessation interventions (hereafter: online cessation interventions) are relatively new innovations and, due to the low costs per smoker, the accessibility (eg, home, work, and public access points), and 24-hour a day availability, have the potential to reach a large proportion of smokers [5]. Internet access is increasing worldwide. In 2016, approximately 94.4% of the Dutch population had access [6] (98.9% of those aged 25 to 45 years, 98.3% aged 45 to 65 years, and 77.6% aged 65 years or older [7]). The level of access for the younger population, aged between 12 and 25 years, was 99.1% in 2015 [7]. Moreover, individuals having access to the Internet in the Netherlands range from 87.0% for the lower educated to 99.5% for the higher educated [7]. However, reach may be a too narrow construct to reflect Internet usage, as activities in use differ [8]. Educational level might, for instance, play a role in online behavior because many individuals may not be sufficiently literate to understand the high literacy level of most written information on the Internet [9]. Yet, a recent study in the Netherlands indicated that usage of eHealth interventions, as recommended, did not differ among education levels. The study also found that eHealth interventions are more often used as recommended to people with a greater need for health care information [10]. As well as Internet access, online cessation interventions allow other opportunities, such as being interactive and tailoring messages to individuals, which has been shown to be effective in changing health behaviors such as enhancing smoking cessation [11]. In addition, due to the low costs per person, online cessation interventions can be cost effective [12]. Therefore, it is relevant to explore the online cessation interventions that are available in the Netherlands and investigate the extent to which these are effective and cost effective.

Cost effectiveness is explored in economic evaluation studies that determine the costs and effects associated with an intervention and compare these with the costs and effects of other interventions or current practice [13]. A typical economic evaluation consists of five steps: (1) identification of relevant costs and effects, (2) measurement of costs and effects, (3) valuation of measured costs and effects, (4) calculation of cost-effectiveness ratio, and (5) sensitivity analysis [14].

### Evaluating Content: Theoretical Basis

Because many online cessation interventions may not have been tested in a randomized controlled trial (RCT), scholars have become interested in evaluating relevant characteristics of behavioral interventions, such as online cessation interventions. The literature shows that the theoretical basis of an intervention and behavior change techniques (BCTs) are characteristics that may influence the impact of behavior [15-17]. Three theories—the social cognitive theory [18], the transtheoretical model [19], and the theory of planned behavior [20]—were particularly important in developing the intervention; their usage in intervention development was associated with increases in effect size [21]. Various sociocognitive theories, including the three mentioned, are integrated in the Integrated Change (I-Change) Model [22-25]. This explains adoption of health behavior in (at least) three phases (ie, awareness, motivation, and action phase), each of which has phase-specific determinants, such as attitudes, social support, and self-efficacy for understanding motivation, and action planning and coping plans to understand the final step from intentions to behavior. Another way to evaluate the content of an intervention is by assessing whether or not the online cessation interventions use specific BCTs that are associated with higher success rates in smoking cessation [26-28]. Five BCTs that are applicable to online interventions and mobile-based interventions have been found to be associated with higher cessation rates [26].These are (1) strengthening exsmoker identity (eg, clear boundaries offered), (2) providing rewards contingent on stopping successfully (eg, use praise or advise on rewarding oneself for moving toward the goal of becoming an exsmoker), (3) advising on changing routines (eg, advising on avoiding cues that will trigger strong urges to smoke), (4) advising and assisting with ways of coping with urges to smoke (eg, develop effective ways of distracting attention from smoking cues in the environment and from urges to smoke), and (5) advising on use of stop-smoking medications (eg, explain the medication available, its benefits, and promote use). The theoretical underpinnings (theoretical factors and BCTs) of interventions, especially those that are not tested in RCTs, may thus provide insights into the content of online cessation interventions (ie, specifying interventions based on relevant theories and BCTs used).

### Objective

Web-based interventions are usually developed for a specific platform, made accessible via computers, tablets, or mobile phones. Not all may be optimized for all platforms. A distinction is made between online interventions (accessed via computer), referred to as eHealth, and mobile-based interventions (apps), referred to as mHealth [29]. Online cessation interventions in this study are defined as online interventions—developed to be accessed via a computer—aimed at enhancing smoking cessation among individual smokers. To explore a broader range of online cessation interventions, combinations of an online intervention and a counselor were deemed relevant. This led to the study goal: to provide an overview of online cessation interventions in the Netherlands and explore their effectivity, cost effectiveness, and theoretical basis.

## Methods

This study used a mixed-methods approach, including (1) a scientific literature search, (2) a grey literature search, and (3) expert input. The methods and the analysis are detailed subsequently.

### Scientific Literature Search

**Figure 1:**
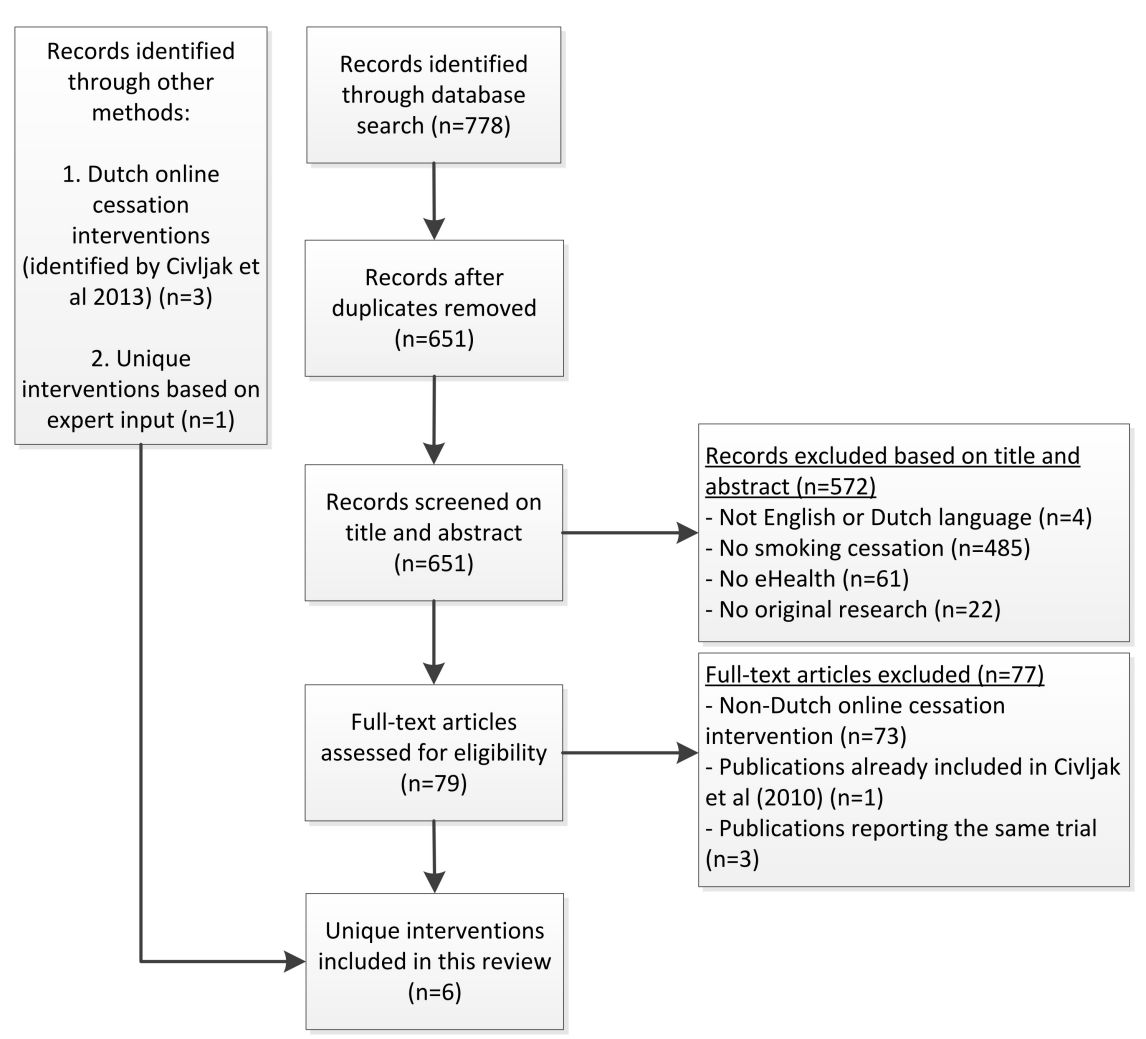
Flowchart of the selection process in the scientific literature.

In the search for online cessation interventions, we used a systematic review of Cochrane [5] regarding the effectiveness of Internet-based interventions, to screen for the Dutch interventions. Because this review was last updated in April 2013, we conducted an additional systematic literature search to explore recent online cessation interventions in the Netherlands up to July 2016. The selection process is depicted in a flowchart (see Figure 1).

The Cochrane review included randomized or quasi-RCTs with smokers that participated in online cessation interventions [5]. All types of online cessation intervention were included; there was no exclusion with respect to method or duration. Also, combinations of interventions were included (when the Internet component was subject to evaluation), if the Internet intervention was an adjunct to behavioral therapy or pharmacotherapy. Trials were excluded if they used Internet solely for recruitment or as a reminder of offline appointments (eg, face-to-face therapy or pharmacotherapy). Moreover, text messaging interventions and mobile-based interventions were not covered. Also, trials with fewer than four weeks of follow-up were excluded.

Similar to the Cochrane review [5], the search strategy for the update of literature was based on the specialized register of the Cochrane Tobacco Addiction Group, including the terms “Internet” or “www” or “web” or “net” or “online” in the title, abstract, or as keywords since 2013. Databases of Cochrane Central Register of Controlled trials (CENTRAL), MEDLINE, and EMBASE were searched via OVID. For full search strategies, see the Tobacco Addiction Group Module [30]. The search led to a total of 651 papers (CENTRAL=96, MEDLINE=163, and EMBASE=392), which two researchers (KLC and BW) screened independently for title and abstract. Inclusion criteria were (1) English or Dutch language, (2) smoking cessation intervention, (3) eHealth (ie, Internet or computer), and (4) original research (excluding study protocols and conference abstracts). Interventions directed at indirect populations (eg, clinicians and nurses) were excluded, as well as lifestyle, telephone, and prevention interventions. The two researchers resolved disagreements through discussion, resulting in 79 potentially relevant papers. The full-text papers were screened by KLC and BW, resulting in exclusion of 10 additional papers. For the relevant Dutch online cessation interventions, the researchers searched through the full-text papers and the Cochrane review [5]. Excluding all non-Dutch intervention studies, the Cochrane review yielded three relevant papers, whereas the updated search (ie, between 2013 and July 2016) yielded six papers. The updated literature search had six papers on Dutch online cessation interventions, from which one was a cost-effectiveness study aiding a study included in the Cochrane review, and four papers were studies on the same intervention (with the same lead author). Due to expert input (described in “Expert Input” section), one intervention was added to the list. Hence, this search led to a final list of six unique online cessation interventions in the Netherlands that were reported in the scientific literature.

### Grey Literature Search

Most online cessation interventions in the Netherlands were not described in peer-reviewed journals. Hence, a Google search was conducted to capture interventions in the grey literature. In line with a previous Dutch study on identifying online tobacco control methods [31], we conducted a Google search to identify actual interventions rather than a search strategy aiming for identifying documents. This is now elaborated in the text. The free-text terms *stoppen met roken* (“smoking cessation”) and *online roken* (“online smoking”) were used, with the intention to capture a broad range of online cessation interventions. For each of the text terms, the first five pages (50 hits) of Google were explored, yielding 100 results (September 25, 2016). These were then screened by two researchers together (KLC and BW) looking at the title (of the page) and content of the site (for potential online cessation interventions), leading to 47 results. Lifestyle interventions, websites targeting indirect groups (eg, clinicians), or telephone-only interventions were excluded. These websites were then screened independently (KLC and BW) for content, including online cessation interventions in the Dutch language. For each website where there was disagreement, consensus was reached via discussion. Here we included a broad range of interventions, including informative static websites and websites promoting any intervention in online form (eg, online counseling). Websites that solely referred to other websites were excluded, as were standalone apps. Six interventions were added due to expert input (described in “Expert Input” section). This led to a final list of 39 online cessation interventions in the Netherlands that were reported in the grey literature.

### Expert Input

To check and complement the previously mentioned searches, Dutch experts in smoking cessation (eg, smoking cessation research and/or online smoking cessation interventions) were recruited using a convenience sampling strategy, in which researchers created a list of potential participants based on personal networks. Twenty experts were approached via email, outlining the goal of the study and asked whether they were able to provide input. Five (25% response rate) provided input for the questionnaire. We asked experts to list the online cessation interventions they knew, adding to the list from the literature search. They provided a list of 21 interventions in total, with seven unique interventions added to the final list (compiled from the literature and grey literature search). One online cessation intervention was added to the scientific literature search, and six to the grey literature search.

### Data Extraction and Analysis

For each included online cessation intervention, two independent reviewers (KLC and BW) systematically extracted data using a predefined extraction form that included general characteristics, four theoretical components (ie, attitudes, social support, self-efficacy, and action planning and coping plans), and five BCTs (ie, strengthening exsmoker identity, providing rewards contingent on stopping successfully, advising on changing routines, advising and assisting with ways of coping with urges to smoke, and advising on use of stop-smoking medications) for the content analysis. Two separate extraction forms were developed (one for scientific literature and one for grey literature), based on literature (theoretical underpinnings) and discussions in the research team. The two reviewers piloted and discussed the extraction form for two interventions, which led to minor adjustments to the data extraction forms.

The extracted data for the interventions identified in the scientific literature included four categories: general study and intervention information; effectiveness, cost effectiveness, and outcome information; theoretical factors; and BCTs. General study and intervention information included intervention name, brief description, and target population. Effectiveness, cost effectiveness, and outcome information included effectiveness (yes, no, and not reported [NR]), effect (in relative risk [RR]), number and percentage abstinent in the intervention condition and in the control condition, control group, outcome measure (for smoking cessation), and cost effectiveness (yes, no, NR). Theoretical factors included were based on the I-Change Model and assessed the presence or absence of the core factors for motivation and behavioral change (yes, no): attitude (discussing advantages and disadvantages of smoking), social influence (addressing social influence or social support), self-efficacy (enhancing one’s capability to become an exsmoker), and action/coping planning (supporting creation or advising on action or coping plans). Literature concerning BCT revealed a large range of potential BCTs that could be selected for reviewing the efficacy of interventions, amounting to 40 different BCTs [21]. Therefore, five BCTs that were especially applicable and relevant to online interventions and mobile-based interventions were included [26]. Consequently, the BCTs included the five BCTs discussed (yes, no): supporting identity change, rewarding abstinence, advising on changing routines, advising on coping, and advising on medication use.

The extracted data for the interventions identified in the grey literature included general intervention information, theoretical factors, and BCTs. General intervention information included intervention name, provider, and target population. The same theoretical factors and BCTs were extracted as for the scientific literature. Websites with only a page of static information about smoking cessation were grouped in the results.

Data were independently extracted by the two researchers and the first author then checked and compared the extracted data. Any disagreements between the researchers were resolved through discussion until consensus was reached [5,26]. Extracted data are presented in tables. Whenever an intervention was shown to be cost effective, this is elaborated in the results section. Information was extracted from the literature and, when available, the researchers checked the online cessation intervention. Suggested interventions by experts are indicated in the tables.

## Results

The mixed-methods yielded 45 unique online cessation interventions (scientific literature: n=6; grey literature: n=39).

### Scientific Literature

The literature revealed six interventions in the Netherlands, which typically targeted adult smokers, who were motivated to quit smoking (thus willing to set a quitting date) (see Table 1). These interventions typically involved tailoring health messages based on the I-Change Model. The exception was the Web-based Attentional Bias Modification (ABM) self-help intervention [32], which aimed to reduce attentional bias and generalize to influence an approach bias for cigarettes and success in quitting. The effectiveness, cost effectiveness, and outcome information of these interventions are detailed in Table 2.

**Table 1:** General study and intervention details of smoking cessation interventions in the scientific literature (n=6).

Intervention	Target population	Brief description
Quit Smoking 2.0 [33]	Smokers (≥18 years, smoker of cigarettes and/or loose-cut tobacco and intending to quit within a year or sooner)	One 7-to-9 page computer-tailored email letter, based on I-Change Model, addressing motivational and postmotivational factors.
Stay Quit For You (SQ4U) [34,35]	Smokers 18-65 years, smoked daily, willing to set a quit date within 1 month, and motivated to quit smoking	The action planning (AP) program provided tailored feedback at baseline and invited respondents to do 6 preparatory and coping planning assignments (the first 3 assignments prior to quit date and the final 3 assignments after quit date). The action planning plus (AP+) program was an extended version of the AP program that also provided tailored feedback at 11 time points after the quit attempt.
Personal Advice in Stopping smoking (PAS) [22,36]	Smokers (Dutch adults) with intention to stop smoking within 6 months	A Web-based computer-tailored smoking cessation intervention with 4 sessions, based on I-Change Model.
Smoke Alert^a^ [37]	Nonsmoker or former smoker; 10 and 20 years; having computer/Internet literacy; having sufficient command of Dutch; no previous exposure to the earlier version of Smoke Alert	Web-based computer-tailored smoking prevention and cessation intervention for adolescents. Based on an intervention shown to be effective [33]. Website intervention providing one-time feedback after filling in a questionnaire.
Support to Quit (STQ) [38-41]	Smokers motivated to stop smoking and aged 18 years	Web-based text and a Web-based video-driven computer-tailored approach for low and high SES smokers, this incorporates 3 to 6 computer-tailored feedback moments with the aim to support smoking cessation.
Attentional Bias Modification (ABM)^a^ [32]	Smokers aged 18-65 years, reporting smoking on a daily basis for at least 1 year and not having made a quit attempt yet	Online 6 sessions of ABM training with the aim to reduce attentional bias and induce more distant generalization effects regarding approach bias and success in quitting.

^a^ Currently available on the Internet.

**Table 2:** Effectiveness, cost effectiveness, and outcome information of smoking cessation interventions in the scientific literature (n=6)

Intervention	Effectiveness	RR (95% CI)	Percentage abstinent, n (%)^a^	Control group	Outcome measure	CE^b^
Quit Smoking 2.0 [33]	Yes	2.48 (1.11-5.55)	Int: 224 (8.5), Con: 234 (3.4)	Nontailored email	7-day point prevalence abstinence at 6 months follow-up	NR
SQ4U [34,35]	Yes	AP+: 1.37 (0.97-1.92); AP: 1.49 (1.07-2.06)	Int (AP+): 53 (7.6), Con: 45 (7.1); Int (AP): 63 (9.0), Con: 45 (7.1)	Questionnaires only	Continued abstinence at 12 months follow-up	NR
PAS [22,36]	Yes	1.50 (0.77-2.94)	Int: 20 (15.2); Con: 12 (10.1)	Usual care	Prolonged abstinence at 12-month follow-up	Yes
Smoke Alert^c^ [37]	NR	NA	NA	NA	NA	NR
STQ [38-41]	Yes	Video: 1.54 (1.08-2.22); text: 1.15 (0.78-1.69)	Int (video): 66 (9.9), Con: 46 (6.4); Int (text): 52 (7.3), Con: 46 (6.2)	Nontailored generic advice	Prolonged abstinence at 12-month follow-up	Yes
ABM^c^ [32]	No	NR, for subgroup: 2.33 (1.14-4.76)	Int (subgroup): 22 (14.2); Con: 10 (6.1)	Placebo-training, continued assessments	Continued abstinence at 6 months follow-up	NR

^a^ Percentage abstinent: n (%) in the intervention condition (Int) and in the control condition (Con).

^b^ CE: Cost effectiveness (yes, no, NR=not reported). If yes, this is detailed in the text.

^c^ Shown effective in Te Poel et al [33].

^d^ Shown effective for subgroup heavy smokers [37].

The effectiveness was shown in four interventions. For Smoke Alert (2.0) [37], the study did not investigate the effectiveness of smoking cessation, but rather investigated and found effects on prevention. Yet, a previous version did show effects on smoking cessation with 17.2% quitters in the control group and 26.8% in the online computer-tailored condition (*P*<.03) [42,43]. Results indicate no overall effectiveness evidence for the ABM intervention except only for heavy smokers. The effective interventions incorporated health messages tailored to responses in an initial questionnaire, which was based on the I-Change Model. Long-term effects were shown in Stay Quit For You (SQ4U) [34,35], Personal Advice in Stopping smoking (PAS) [22,36], and Support to Quit (STQ) [38-41], using a 12-month follow-up with either continued or prolonged abstinence outcome measure. For these interventions with long-term follow-ups, we conducted a meta-analysis in which the Mantel-Haenszel fixed-effect model was used to pool the effectiveness of these interventions. This resulted in a pooled RR of 1.39 (95% CI 1.18-1.65). For two interventions (PAS and STQ), studies also found support for its cost effectiveness from a societal perspective. Using prolonged abstinence as the outcome measure, the cost-effectiveness study for PAS showed that the mere multiple computer-tailored program had the highest probability of being cost effective as in this group €5100 had to be paid for each additional abstinent participant (compared to usual care) [22]. Using prolonged abstinence, the study for STQ indicated that with a willingness to pay €1500, the video-based intervention was likely to be the most cost-effective treatment. For each additional abstinent participant, €1500 had to be paid within the video-based intervention [38]. Given the fact that, in the Netherlands, cost effectiveness is concluded when the additional costs (for an additional quality-adjusted life year [QALY]) are between €20,000 and €80,000, the conclusion is that these interventions were highly cost effective [44]. Normally, this threshold is dependent on the severity of the disease. In prevention, a threshold of €20,000 is often considered [45].

The interventions were also evaluated and analyzed with regard to the theoretical factors and BCTs used (see Table 3). For the theoretical factors, all interventions (with one exception) addressed attitudes, social influence, self-efficacy, and action or coping planning (83%, 5/6). The exception was the ABM intervention [32], which had a different theoretical basis, namely retraining implicit associations (automatic attentional processes). Attentional bias is the tendency of certain cues in a person’s environment to attract and/or hold the individual’s attention preferentially, such as cigarettes. This attentional bias could be retrained toward or away from the substance-related cues [32]. The SQ4U [34,35] and STQ [38-41] interventions included all BCTs, whereas half of the interventions included all BCTs, except rewarding abstinence (50%, 3/6).

**Table 3:** Theoretical factors and behavior change techniques (BCTs) of smoking cessation interventions in the scientific literature (n=6).

Intervention	Attitude	Social influence	Self-efficacy	Action & coping planning	BCTs^a^				
					1	2	3	4	5
SQ4U^b^ [34,35]	Yes	Yes	Yes	Yes	Yes	Yes	Yes	Yes	Yes
PAS^b^ [22,36]	Yes	Yes	Yes	Yes	Yes	NR	Yes	Yes	Yes
Smoke Alert [37]	Yes	Yes	Yes	Yes	Yes	Yes	Yes	Yes	Yes
Quit Smoking 2.0^b^ [33]	Yes	Yes	Yes	Yes	Yes	No	Yes	Yes	Yes
STQ^b^ [38-41]	Yes	Yes	Yes	Yes	Yes	Yes	Yes	Yes	Yes
ABM^b^ [32]	No	No	No	No	No	No	No	No	No
Percentage; n (%)^c^	5 (83)	5 (83)	5 (83)	5 (83)	5 (83)	3 (50)	5 (83)	5 (83)	5 (83)

^a^ BCT1: Supporting identity change; BCT2: rewarding abstinence; BCT3: advising on changing routines; BCT4: advising on coping; and BCT5: advising on medication use.

^b^ Mentioned by experts.

^e^ Percentage (%) interventions of scientific literature including this factor/BCT.

### Grey Literature

The grey literature revealed 39 interventions in the Netherlands, which typically were targeted at smokers (see Table 4). The online cessation interventions were grouped as websites providing only static information (n=23) and websites incorporating an interactive component (n=17). Seven unique interventions were added due to expert input (see Tables 3 and 4). Some experts indicated that it was difficult to evaluate the effectiveness of the online cessation interventions, with the exception of a few reported in scientific literature. The grey literature yielded a variety of online cessation interventions with different providers, but only minimal research has been conducted to test their effects, resulting in little scientific support for their evidence. One intervention was identified in both the grey literature and in the scientific literature (Smoke Alert [37], which—as outlined previously—was effective in preventing the initiation of smoking among adolescents [37]). An intervention (Online zelfhulp tabak [46]) identified in the grey literature search and via expert input was reported to be effective; it was to be tested in a RCT. After contacting the provider of the intervention, it was revealed that the claim on effectiveness was based on a trial of a similar intervention on alcohol [47]. It was stated that ‘’due to the generalizability of the self-help module, the self-help tobacco module was recognized as well.” Hence, the quality of the interventions identified in the grey literature search seems to lack scientific basis because the number of effectiveness studies is limited.

**Table 4:** General intervention information of grey literature (translation in brackets).

Intervention	Provider	Target population	Static?
De StopSite (The QuitSite)^a^	Luchtsignaal	Smokers aged ≥18 years	Interactive
uQuit.nl^a^	Universiteit Nijmegen, VU Amsterdam, and IVO	Smokers / student smokers	Interactive
Tabakstop (Tobaccostop)	Stichting tegen kanker	Smokers	Interactive
ExSmokers (iCoach)^a^	European Commission	Smokers	Interactive
Stoppen met roken (Smoking cessation)	Pfizer	Smokers	Interactive
Roken de Baas (Boss of your smoking)^a^	Tactus Verslavingszorg	Smokers	Interactive
CZ Stoppen met roken coach (CZ smoking cessation coach)^a^	CZ	Smokers	Interactive
Stoppen met roken (Smoking cessation)^a^	Minddistrict	Smokers	Interactive
StopExpert^a^	ExaCare	Smokers	Interactive
Online zelfhulp tabak (Online self-help tobacco)^a^	Jellinek	Smokers	Interactive
Stoppen met roken, in één dag van het roken af (Smoking cessation, quit in one day)^a^	De opluchting	Smokers	Interactive
Wat doe je om te stoppen met roken? De PZP helpt (What do you do to quit smoking? PZP helps)	PZP	Smokers with an insurance for the police (PZP)	Interactive
Home Roken—Ja (Home Smoking—Yes)	Victas	Smokers	Interactive
Training stoppen met roken—Kentra (Training smoking cessation—Kentra)	Kentra	Smokers	Interactive
stoppen met roken (smoking cessation) | iLifecoach	iLifeCoach	Smokers	Interactive
Online cursus stoppen met roken | Zo stop je wel (Online course smoking cessation | You’ll quit like this)	Zostopjewel.nl	Smokers	Interactive
Stoppen met roken (Smoking cessation)	Gezondheidsnet.nl	Smokers	Static
ikstopnu.nl (Iquitnow.nl)^a^	Ikstopnu.nl	Smokers	Static
Ik stop! (I quit!)	Ikstop.nl	Smokers	Static
NuStoppenmetRoken.nl-Stoppen met Roken (Quitsmokingnow.nl—Smoking cessation)	NuStoppenmetRoken.nl	Smokers	Static
Stoppen met roken (Smoking cessation)	Nederlands Huisartsengenootschap	Smokers	Static
Stoppen met roken (Smoking cessation)	Medical Media BV	Smokers	Static
Hoe kan ik stoppen met roken? (How can I quit smoking?)	Stichting Opvoeden.nl	Smokers	Static
Rokeninfo.nl (Smokinginfo.nl)^a^	Trimbos Instituut	Smokers	Static
Ex rokers (Former smokers)	Ex rokers	Smokers	Static
Tips stoppen met roken (Tips to quit smoking)	Christelijke Mutualiteit	Smokers	Static
Hoe kan ik stoppen met roken? (How to quit smoking?)	GGD	Smokers	Static
Ik Wil Stoppen Met Roken.NU (I Want To Quit Smoking.NOW)^a^	Ik Wil Stoppen Met Roken.NU	Smokers	Static
Welkom bij de stoppen met roken test! (Welcome to the smoking cessation test)^a^	ProStop	Smokers	Static
soChicken	soChicken	Smokers	Static
Waarom stoppen met roken? (Why quit smoking?)—Watchtower ONLINE LIBRARY	Watchtower	Jehovah’s Witnesses	Static
Stoppen met Roken (Smoking cessation)	Verslaving.nu	Smokers	Static
Stoppen met Roken? (Quit smoking?)	Dokteronline.com	Smokers	Static
Stoptober^a^	Stoptober^b^	Smokers	Static
Stoppen met Roken.nl (Smoking Cessation.nl)	Stichting stop bewust	Smokers	Static
Stoppen met roken-GGD Fryslan (Smoking cessation—CHS of Fryslan)	GGD	Smokers	Static
Welkom bij nl.support.stop-met-roken (Welcome to nl.support smoking cessation)	NSSMR	Smokers	Static
Stoppen met roken (Smoking cessation)—YouTube	Stichting Gezondheid	Smokers	Static
Eenrookvrijleven.nl (Smokefreelife.nl)^a^	Eric Eraly	Smokers	Static

^a^Mentioned by experts.

^b^Stoptober: KWF Kankerbestrijding, Hartstichting, Longfonds, Ministerie van Volksgezondheid, Welzijn en Sport (VWS), Trimbos Instituut, GGD GHOR Nederland, and Alliantie Nederland Rookvrij.

The interventions were extracted on theoretical factors and BCTs (see Multimedia Appendixes 1 and 2) with the exception of those that were not accessible to the researchers. This was especially the case for interactive interventions, with tailored messages or combined with feedback from a counselor. For those that could be evaluated, the results show that at least 67% (26/39) addressed attitude, mentioning the advantages and disadvantages of smoking cessation. Many interventions also addressed social influence and action or coping planning (at least 44%, 17/39 and 51%, 20/39, respectively), by advising on social support, planning to continue to abstain from smoking, or dealing with difficult situations. At least 31% (12/39) of the interventions addressed the self-efficacy of smokers by persuasion or via modeling, enhancing beliefs that the smoker is able to become an exsmoker. Regarding BCTs, most online cessation interventions provided information and advice on the importance of changing routines (at least 49%, 19/39) and medication usage (at least 54%, 21/39). A moderate number of interventions supported identity change (at least 28 %, 11/39), provided tips or provided rewards for abstinence (at least 38%, 15/39), and advised on coping strategies (at least 44%, 17/39).

## Discussion

This study provides an overview of online cessation interventions in the Netherlands, up to 2016. They are broadly categorized in two lists: interventions reported in the scientific literature and those identified in the grey literature. Expert input overlapped with the lists and added a few unique interventions.

### Summary of Evidence

The first goal of this study was to assess the effectiveness of online interventions concerning smoking cessation as defined by the most strictly reported outcome measure. Our search revealed that six online cessation interventions in the Netherlands were investigated for their effectiveness in trials. With one exception, these were all interactive individually tailored interventions based on sociocognitive models (ie, the I-Change Model). Smokers filled in questionnaires dealing with demographics, smoking behavior, and sociocognitive constructs. The answers were used to yield tailored motivational messages. The exception was the ABM intervention, which focused on reducing attentional bias and inducing more distant generalization effects regarding approach bias and success in quitting [32]. The effects indicated that smokers using an online cessation intervention are 1.15 to 2.84 times more likely to become a former smoker compared to the control condition. This range and the meta-analysis (with a pooled RR 1.39, 95% CI 1.18-1.65) seem in line with the Cochrane review with a pooled RR (Mantel-Haenszel fixed-effect) of 1.41 for computer-tailored online smoking cessation interventions (95% CI 1.11-1.78) [5]. These reported effects of online smoking cessation interventions may perhaps be underestimated because conservative analyses were used, including penalized imputation which categorizes missing data as smoking. A recent study showed that penalized imputation biases the reported effects of online smoking cessation interventions, favoring the condition with the lowest proportion of missing data [48]. Interestingly, many more interventions were identified using grey literature; yet, none of them were evaluated in terms of their effectiveness.

A second goal was to identify the cost effectiveness of online cessation interventions in the Netherlands. Of the six interventions identified, two were tested and shown to be cost effective, meaning that the costs for an additional quitter (or QALY) did not surpass a conservative threshold of €20,000. PAS was cost effective, with costs of €5100 for each additional quitter [22], and STQ with its tailored video-based intervention was cost effective with costs of €1500 per additional quitter [38]. When the additional costs for an additional QALY is between €20,000 and €80,000, an intervention is considered to be cost effective in the Netherlands. Normally, this threshold is dependent on the severity of the disease. In prevention, often a threshold of €20,000 is considered [45]. There is, however, no cut-off point with regard to smoking abstinence rates as outcome, which suggests future research should identify an acceptable cut-off point for the willingness to pay per abstinent participant [49]. Extracting data on theoretical components and BCTs revealed that these I-Change Model-based interventions all addressed attitudes, social influence, self-efficacy, and action or coping planning. It also revealed that these interventions support identity change, and advise on changing routines and on coping. Because not all interventions were available to the public or researchers, there is some uncertainty about the percentage interventions rewarding abstinence and advising on medication. Other interventions that have potential to be implemented are SQ4U (scoring high on all BCTs and shown to be effective) [34] and Quit Smoking 2.0 (shown to be effective and recommended based on quality, practicability, and effectiveness on loketgezondheid.nl) [33]. However, data concerning their cost effectiveness are lacking, and for Quit Smoking 2.0 the follow-up is 6 months only [33]. Hence, because a follow-up of 12 months indicates long-term effects, SQ4U [34,35] and especially PAS [22,36] and STQ [38-41] appear to be promising interventions for implementation in the Netherlands. The effective interventions (ie, Quit Smoking 2.0, SQ4U, PAS, and STQ), are no longer publicly available because they were part of a research study. Yet, if organizations are interested, they can be obtained via Vision2Health (a health consultancy initiative offering evidence-based health promotion interventions). For instance, Quit Smoking 2.0 has been offered by several regional health authorities on request. The youth intervention Smoke Alert is still online because it is offered by Trimbos and has received approximately 13,000 unique visitors. Interestingly, it was shown that such online interventions did not differ in usage as recommended by individuals with different educational levels [10]. This indicates the potential to be effectively implemented for smokers with different levels of education [50].

A third goal was to assess the theoretical underpinnings of the interventions. The majority of the interventions that were also evaluated for their effectiveness and cost effectiveness used most, if not all, of the theoretical factors and the five BCTs. The interventions identified from the grey literature also used some of these factors and techniques. Some appeared to have included several, from which one might conclude that they are more likely to be successful. They could be candidates for testing in RCTs. But the theoretical factors and BCTs have to be applied according to parameters, which impact on the degree of effectiveness [51].

In conclusion, it would be beneficial to many smokers, and even cost effective from a societal perspective, if effective and cost-effective interventions were to become available to the public after completion of a RCT study. For public health impact, it is important that these evidence-based interventions are not only available online, with no marketing (passive availability), but that they be promoted proactively, involving marketing to various stakeholders within health care (eg, general practitioners, dental practices, and other primary health care providers) and public health organizations (eg, regional health education authorities). Moreover, it is important for universities and research institutes to consider implementation of the intervention and to create a business model [52]. Researchers need to include this step, enhancing availability and usage of the developed intervention. Furthermore, more funding is needed to implement interventions that are proven to be effective and cost effective, in order to enhance usage and availability of those already online, such as PAS [22,36] and STQ [38-41]. In addition, future research may also look at the cost effectiveness to bridge the gap between research and practice because cost-effectiveness studies provide a financial argument for investment in effective interventions, such as SQ4U [34,35]. Many interventions were identified in the grey literature, more than in the scientific literature. Some may be promising if they address theoretical factors and BCTs, but they lack scientific support for effectiveness. This may be an argument for introducing a quality mark to reflect the extent of the scientific evidence. Providers could then submit their intervention for evaluation. Such a mark is important for health care stakeholders and smokers to identify quality online cessation interventions.

### Limitations

This study has several limitations. One is the lack of an evaluation tool to investigate the quality or effectiveness of nontested reports identified in the grey literature. To evaluate the content, we assessed whether the interventions implemented relevant theoretical components and BCTs, identified as being associated with smoking cessation [21,26-28]. However, BCTs have limitations because they simplify reality [51]. They are generic and their application varies. Current practice often does not recognize that these BCTs have parameters for effectiveness and that methods can interact with one another. Context factors may also impact the effectiveness of BCTs [51]. Hence, the evaluation of whether interventions include BCTs may result in overly simplistic or even incorrect conclusions about the quality of the identified interventions. As well as the evaluation of the content via BCTs, the delivery, such as engagement and ease-of-use features, is important [26]. Delivery evaluation was not included in this study. Similarly, previously mentioned criticism of BCTs also applies with regard to the inclusion of theoretical factors in the interventions. This study is cautious in its recommendations based on the inclusion of theoretical factors and BCTs. Another limitation of the study may be the limited generalizability of results to other countries. Findings reflect the online cessation interventions in the Netherlands; other countries may have implemented and tested different interventions. It would, therefore, be interesting to conduct similar studies in other countries and compare potential differences. Moreover, no risk of bias assessment (eg, a quality checklist) was conducted, which may have over- or underemphasized the strength of the evidence of some studies when calculating the pooled effect. Yet, our estimates were in line with others reported in, for example, the Cochrane review on online cessation interventions [5]. Nevertheless, study results should be interpreted with some caution.

The usage of mobile phones in everyday life is becoming increasingly important, indicating that online cessation interventions (developed to be accessed via a computer) may also need to function on a mobile platform. In the most recent Cochrane review on mobile-based smoking cessation interventions, only five studies were included [53]. None of them was developed for the Dutch population; results were heterogeneous with three of five interventions crossing the line of no effects. Hence, more research is required to explore opportunities for mobile-based interventions by investigating effectiveness and cost effectiveness. The lack of Dutch mobile phone-based interventions—tested for effectiveness—indicates the need to transfer the effective online cessation interventions to the mobile phone. Whereas mHealth interventions may have great potential, a recent Dutch study found that eHealth was more effective in realizing physical activity [54]. This does not imply that mHealth cannot be effective, but rather that we need to identify how to use the two modalities optimally. The potential of mHealth, as well as issues such as effectiveness, cost effectiveness, and usability [55,56], should be topics for future research, as well as exploring ways of improving computer tailoring of health messages (via different algorithms) [57].

### Conclusions

This study provides an overview of Dutch online cessation interventions, while assessing their effectivity, cost effectivity, and theoretical basis. This mixed-methods study may also serve as a vantage point for future overviews in other countries. Although the Internet may well provide the possibility of offering various smoking cessation interventions, the user in the Netherlands is left bewildered about its efficacy because most of the data are not available or are not offered to the smokers. If the Internet wants to live up to its promise, clear regulations governing effectiveness of interventions have to be devised to avoid disappointment and failed quit attempts. Policy regulations may be needed to regulate the proliferation of interventions and foster quality. As nonadoption of cost-effective eHealth interventions is both detrimental at the micro level (smokers not profiting from effective interventions) as well as the macro level (unnecessary high costs of smoking due to nonimplementation of effective methods), reasons for nonadoption, as well as strategies enhancing such an adoption, are a prerequisite to ensure a significant public health impact of effective eHealth and mHealth interventions.

## Appendix

Multimedia Appendix 1 Theoretical factors of grey literature.

Multimedia Appendix 2 BCTs of grey literature.

## Abbreviations

ABM: Attentional Bias Modification
BCT: behavior change technique
PAS: Personal Advice in Stopping smoking
QALY: quality-adjusted life year
RCT: randomized controlled trial
RR: relative risk
SQ4U: Stay Quit for You
STQ: Support to Quit
VWS: Volksgezondheid, Welzijn en Sport
