# Supplementary material for: A Review of the Theoretical Basis, Effects, and Cost Effectiveness of Online Smoking Cessation Interventions in the Netherlands: A Mixed-Methods Approach
Source: J Med Internet Res. 2017 Jun 23;19(6):e230. doi: 10.2196/jmir.7209 (PMC5501927; doi:10.2196/jmir.7209)
Supplement: Multimedia Appendix 1 [file jmir_v19i6e230_app1.pdf]

Multimedia Appendix 1. Theoretical factors of grey literature

Intervention	Static	Effectivity	Attitude	Social influence	Self-efficacy	Action & Coping planning
De StopSite (The QuitSite)[a]	Interactive	NR	Yes	Yes	Yes	Yes
uQuit.nl[a]	Interactive	NR	Yes	Yes	Yes	Yes
Tabakstop (Tobaccostop)	Interactive	NR	Yes	Yes	Yes	Yes
ExSmokers (iCoach)	Interactive	NR	Yes	No	Yes	Yes
Stoppen met roken (Smoking cessation)	Interactive	NR	Yes	Yes	No	Yes
Roken de Baas (Boss of your smoking)[a]	Interactive	NR	Yes	Yes	No	Yes
CZ Stoppen met roken coach (CZ smoking cessation coach)[a]	Interactive	NR	Yes	NR	NR	NR
Stoppen met roken (Smoking cessation)[a]	Interactive		NR	NR	NR	NR
StopExpert[a]	Interactive	NR	NR	NR	NR	NR
Online zelfhulp tabak (Online selfhelp tobacco)[a]	Interactive	NR	NR	NR	NR	NR
Stoppen met roken, in één dag van het roken af (Smoking cessation, quit in one day)[a]	Interactive	NR	NR	NR	NR	NR
Wat doe je om te stoppen met roken? De PZP helpt (What to do to quit smoking? PZP helps)	Interactive	NR	NR	NR	NR	NR
Home Roken – Ja (Home Smoking – Yes)	Interactive	NR	NR	NR	NR	NR
Training stoppen met roken – Kentra (Training smoking cessation – Kentra)	Interactive	NR	NR	NR	NR	NR
stoppen met roken (smoking cessation) | iLifecoach	Interactive	NR	NR	NR	NR	NR
Online cursus stoppen met roken | Zo stop je wel (Online course smoking cessation | You'll quit like this)	Interactive	NR	NR	NR	NR	NR
Stoppen met roken (Smoking cessation)	Static	NR	Yes	Yes	Yes	Yes
ikstopnu.nl (Iquitnow.nl)[a]	Static	NR	Yes	Yes	Yes	Yes
Ik stop! (I quit!)	Static	NR	Yes	Yes	Yes	Yes
NuStoppenmetRoken.nl - Stoppen met Roken (Quitsmokingnow.nl – Smoking cessation)	Static	NR	Yes	Yes	Yes	Yes
Stoppen met roken (Smoking cessation)	Static	NR	Yes	No	Yes	Yes
Stoppen met roken –(Smoking cessation)	Static	NR	Yes	Yes	No	Yes
Hoe kan ik stoppen met roken? (How can I quit smoking?)	Static	NR	Yes	Yes	Yes	Yes
Rokeninfo.nl (Smokinginfo.nl)[a]	Static	NR	Yes	Yes	No	Yes
Ex rokers (Former smokers)	Static	NR	Yes	Yes	Yes	Yes
Tips stoppen met roken (Tips to quit smoking)	Static	NR	Yes	Yes	No	Yes
Hoe kan ik stoppen met roken? (How to quit smoking?)	Static	NR	Yes	Yes	No	Yes

Ik Wil Stoppen Met Roken.NU (I Want To Quit Smoking.NU (NOW))[a]	Static	NR	Yes	No	No	Yes
Welkom bij de stoppen met roken test! (Welcome to the smoking cessation test)[a]	Static	NR	Yes	No	Yes	Yes
soChicken	Static	NR	Yes	No	No	Yes
Waarom stoppen met roken? (Why quit smoking?) — Watchtower ONLINE LIBRARY	Static	NR	Yes	Yes	No	No
Stoppen met Roken (Smoking cessation)	Static	NR	Yes	No	No	No
Stoppen met Roken ? (Quit smoking?	Static	NR	Yes	No	No	No
Stoptober[a]	Static	NR	NR	Yes	NR	NR
Stoppen met Roken.nl (Smoking Cessation.nl)	Static	NR	No	No	No	No
Stoppen met roken - GGD Fryslan (Smoking cessation – CHS of Fryslan)	Static	NR	Yes	No	No	No
Welkom bij nl.support.stop-met-roken (Welcome to nl.support smoking cessation)	Static	NR	No	No	No	No
Stoppen met roken (Smoking cessation) – YouTube	Static	NR	Yes	No	No	No
Eenrookvrijleven.nl (Smokefreelife.nl)[a]	Static	NR	NR	NR	NR	NR
**Percentage**[a]			67	44	31	51

a = Percentage (%) interventions of grey literature including this theoretical factor
